# Huge Fungating Benign Phyllodes Tumor of Breast

**DOI:** 10.12669/pjms.343.15548

**Published:** 2018

**Authors:** Altaf Hussain Rathore

**Affiliations:** 1 Prof. Dr. Altaf Hussain Rathore, FRCS. Retired Professor of Surgery, Punjab Medical College, Faisalabad, Pakistan

**Keywords:** Huge fungating, Phyllodes tumor, Benign

## Abstract

A 25 years old unmarried woman was operated for a huge fungating tumor of right breast, proved to be a benign phyllodes tumor by preoperative and post-operative biopsy by two different renowned histopathology laboratories. Simple mastectomy with excision of wide margin of skin and pectoral major was done. She is followed up for 10 months; she has a good scar and no sign of any secondary so far.

## INTRODUCTION

Phyllodes tumor is a rare biphasic neoplastic lesion of the breast composing of stromal and epithelial components.^1^ Its incidence is 0.3-1% of all the breast tumors.^2^ It was first described by the name cystosarcoma phyllodes by Mullor which was labeled to the present name by WHO classification in 1982.^3^,^4^ It is divided into benign and malignant depending upon stromal and atypical cells.^5^ About 70-90% of tumors are benign. Their recurrence rate is 5-15%.^6^ The usual age of occurrence is 40-50 years, though cases as young as 14 years with phallodes tumors have also been reported.^7^ As far as malignant tumors are concerned, they metastasize locally or by blood to bones and lungs^8^ but lymphatic spread is rare.^9^ We present a rare fungating phyllodes tumor of the breast in a young unmarried female which has been proved to be benign by repeated biopsy by different laboratories.

## CASE REPORT

A 25 years old unmarried female developed a painless, firm, round, mobile swelling in right breast since one year which was growing in size progressively. It was incised by a quack under local anesthesia three month back, which started growing with a great speed since that period.

**Fig 1:**
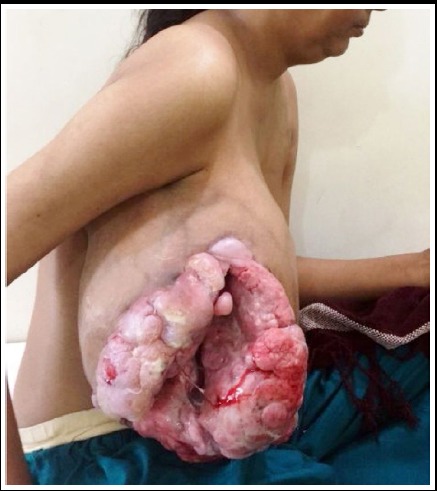
Pre-operative lateral view of fungating tumor.

**Fig 2:**
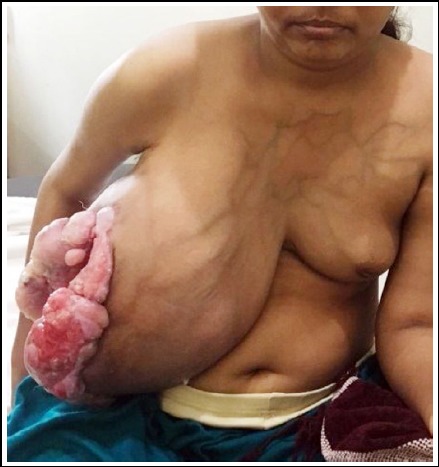
Preoperative frontal view.

It was hanging from the chest, painless, fungating (Fig. 1 and 2), discharging offensive fluid. Its size was 30x25cms. It was hindering in performing her daily routine jobs. She was grossly anemic and clinically there were no glands in axilla. X-ray chest was clear; Hb was 6g/dl. There was no history of any breast ailment in the family. Preoperative open biopsy was taken which revealed benign phyllodes tumor on histopathology. The tumor had a loose adhesion with the pectoral major.

## OPERATION

**Fig 3:**
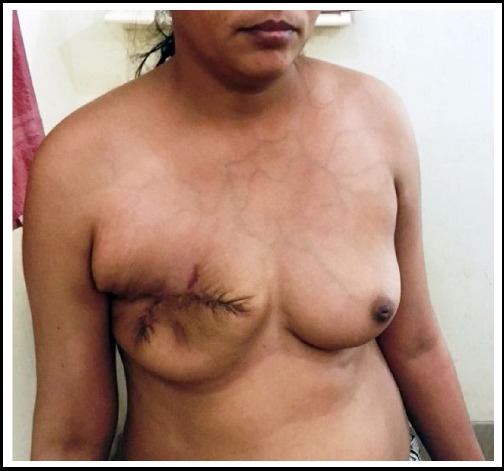
Post-operative (7^th^ month) view.

Preoperative six units of blood were transfused. The patient was operated under general anesthesia. Simple mastectomy with excision of pectoralis major muscle and wide excision of the edges was done. A primary repair was performed. Patient was discharged three days after operation. Sutures were removed on 9th post-operative day. She had uneventful recovery and healing. The weight of removed breast was 5 kg. Post-operative biopsy report was also benign phyllodes tumor. Patient last reported 10 months after the operation (Fig.3). There was no apparent local or distant recurrence.

## DISCUSSION

Phyllodes tumor is a rare tumor of breast which is diagnosed by a typical history of a rounded painless, mobile and fast growing swelling of breast. It can grow into a huge size within weeks. Clinically it is very difficult to distinguish between benign and malignant variety.^10^ That is why excision of 2cms of tissue beyond edges is recommended during surgery to avoid recurrence. Surgery is the gold standard treatment of the tumor, even of malignant ones as radiotherapy, chemotherapy and hormonal therapy have no role in treatment of this tumor. Though usual age is 40-50 years but our case was young female of 25 years. This is a rare case that it was fungating and attained a huge size yet it was not malignant, which proves this point that very big, even fungating phyllodes may not be necessarily a malignant, so primary treatment should be local surgery with wide margins of skin.
